# Cortical morphological markers in children with autism: a structural magnetic resonance imaging study of thickness, area, volume, and gyrification

**DOI:** 10.1186/s13229-016-0076-x

**Published:** 2016-01-25

**Authors:** Daniel Y.-J. Yang, Danielle Beam, Kevin A. Pelphrey, Sebiha Abdullahi, Roger J. Jou

**Affiliations:** Center for Translational Developmental Neuroscience, Child Study Center, Yale University, New Haven, CT USA

**Keywords:** Autism spectrum disorder, Neuroanatomy, Surface-based morphometry, Brain development, Brain structure

## Abstract

**Background:**

Individuals with autism spectrum disorder (ASD) have been characterized by altered cerebral cortical structures; however, the field has yet to identify consistent markers and prior studies have included mostly adolescents and adults. While there are multiple cortical morphological measures, including cortical thickness, surface area, cortical volume, and cortical gyrification, few single studies have examined all these measures. The current study analyzed all of the four measures and focused on pre-adolescent children with ASD.

**Methods:**

We employed the FreeSurfer pipeline to examine surface-based morphometry in 60 high-functioning boys with ASD (mean age = 8.35 years, range = 4–12 years) and 41 gender-, age-, and IQ-matched typically developing (TD) peers (mean age = 8.83 years), while testing for age-by-diagnosis interaction and between-group differences.

**Results:**

During childhood and in specific regions, ASD participants exhibited a lack of normative age-related cortical thinning and volumetric reduction and an abnormal age-related increase in gyrification. Regarding surface area, ASD and TD exhibited statistically comparable age-related development during childhood. Across childhood, ASD relative to TD participants tended to have higher mean levels of gyrification in specific regions. Within ASD, those with higher Social Responsiveness Scale total raw scores tended to have greater age-related increase in gyrification in specific regions during childhood.

**Conclusions:**

ASD is characterized by cortical neuroanatomical abnormalities that are age-, measure-, statistical model-, and region-dependent. The current study is the first to examine the development of all four cortical measures in one of the largest pre-adolescent samples. Strikingly, Neurosynth-based quantitative reverse inference of the surviving clusters suggests that many of the regions identified above are related to social perception, language, self-referential, and action observation networks—those frequently found to be functionally altered in individuals with ASD. The comprehensive, multilevel analyses across a wide range of cortical measures help fill a knowledge gap and present a complex but rich picture of neuroanatomical developmental differences in children with ASD.

**Electronic supplementary material:**

The online version of this article (doi:10.1186/s13229-016-0076-x) contains supplementary material, which is available to authorized users.

## Background

Autism spectrum disorder (ASD) is a highly prevalent [1], strongly genetic [2], neurodevelopmental disorder, characterized by social and communication impairments as well as repetitive behaviors and restricted interests [3]. Decades of neuroimaging research in ASD have been informative in the search for its complex neurobiology. Numerous fMRI studies have consistently identified atypical brain functioning in a number of regions commonly referred to as the social brain [4–6], including multiple regions within the frontal [7, 8] and temporal cortices [9–12]. Of particular interest is the right posterior superior temporal sulcus (pSTS), which is a central node in social information processing and is functionally disrupted in ASD [6]. On the other hand, the search for neuroanatomical abnormalities via non-diffusion-weighted, structural MRI (sMRI) has yielded less consistent findings, aside from a tendency towards early brain overgrowth during the first years of life [13–16]. The field has yet to identify reliable cortical neuroanatomical markers for ASD using sMRI [17–19]. Moreover, recent studies suggest that neuroanatomical abnormalities in ASD are highly age-dependent [20, 21]. Prior structural neuroimaging research in ASD has included mostly older individuals (adolescents and adults) [22–32], which may not depict an accurate picture about pre-adolescent children with ASD. Among the small number of studies that included children with ASD, many samples consisted of only young children [13, 33, 34], which also may not provide a full picture of the neuroanatomical developmental trajectories in ASD more broadly across childhood. The primary objective of the current study is to address this gap in knowledge by charting cortical developmental differences in a large sample of pre-adolescent children with ASD 4–12 years of age.

We employed a standard processing pipeline of the widely-employed, freely-available FreeSurfer image analysis suite [35] (http://surfer.nmr.mgh.harvard.edu/), which uses surface-based morphometry (SBM), as opposed to voxel-based morphometry (VBM), that reconstructs and characterizes key information about brain structure. It provides better alignment of cortical landmarks than volume-based registration and does not result in an age-associated bias between older and younger children when registering children’s brains to a common space [36]. These qualities make FreeSurfer an ideal tool for accurately comparing the brain anatomy of pre-adolescent children.

By reconstructing the brain using spatially fine-grained 3D surface meshes via vertices [37–39], FreeSurfer offers a wide range of cortical structural measures, including cortical thickness (CT), surface area (SA), cortical volume (CV), and cortical gyrification (CG). These measures are correlated with different aspects of cerebral cortical microstructure and are briefly reviewed here. By definition, CV is the product of its two main constituents: CT and SA. It has been widely documented that neurons within the cerebral cortex are modularly organized into ontogenetic columns perpendicular to the surface of the brain [40]. According to the radial unit hypothesis [41], CT is determined by the number of neurons within a column, whereas SA is determined by the number of columns. Furthermore, CT and SA are believed to be determined by different types of progenitor cells [42]. According to the intermediate progenitor hypothesis [43], intermediate progenitor cells (IPC) produce only neurons through symmetric division at subventricular and intermediate zones; the newborn neurons migrate along radial glial fibers to their final cortical destinations and form ontogenetic columns arranged as radial units. The IPCs as neurogenic transient amplifying cells of the developing cerebral cortex amplify each radial unit to form CT but not SA. In contrast, according to the radial unit hypothesis [41], the early proliferation of radial unit progenitor cells (neuroepithelial founder cells and radial glia, which produce single IPCs through asymmetric division at the apical surface) leads to an increase in the number of ontogenetic columns and thus the size of SA. The subdivision of CV into CT and SA thus allows the evaluation of whether CV differences in ASD [44, 45] are attributable to CT, SA, or both, which in turn provides the basis for further investigation of the corresponding cellular and genetic [46] mechanisms underlying ASD.

Furthermore, CG (also known as gyrus formation and cortical folding) is thought to reflect how the human brain manages the problem of packing a phylogenetically increasing cortical surface area into the limited space of the cranial vault [47, 48]. The question of why the cerebral cortex folds the way it does has puzzled the field for a long time. There are a number of interacting, non-mutually exclusive hypotheses of cortical folding in the literature [49]. One of the most influential views is the axonal tension hypothesis [50], which posits that axonal tension between cortical regions induces folding by pulling on the cortex, which draws together regions that are strongly connected and creates folding. This hypothesis is supported by the close relationship between CG and greater local neural connectivity [48, 51]. In contrast, the differential tangential expansion hypothesis [52, 53] postulates that the outer, superficial cortical layers expand more rapidly than the inner, deep cortical layers, which causes cortical folding. Still, a recent synthesized viewpoint [49] suggests that the radial intercalation of young cortical neurons into the outer layer of the developing cortical plate may cause the cortical plate to expand tangentially more rapidly than the underlying tissue, which in turn leads to cortical folding. It is currently unclear which of these hypotheses is more correct, while each of these hypotheses may generate new directions of investigation about CG development in ASD. In individuals with ASD, CG has been reported to be altered in several areas including the frontal [51, 54, 55] and temporal-occipital regions [56].

Because all of the four cortical measures likely provide unique and complementary information about brain structure and are associated with differential genetic and cellular mechanisms in the brain, it is important to include all of them in the same study of ASD, as a more comprehensive picture is likely to emerge. To our knowledge, no study to date has examined all of these four measures in a predominantly pre-adolescent group of children with ASD. For this reason, we used SBM and examined CT, SA, CV, and CG in one of the largest samples of pre-adolescent children with ASD and a group of typically developing (TD) children who were well-matched on gender, age, and IQ. With the large sample and a comprehensive set of vertex-based cortical morphometric measures, we aim to better understand the neurodevelopmental brain structural differences in ASD during childhood.

## Methods

### Participants

Study participants included 101 children (all males) between 4 and 12 years of age. They consisted of 60 boys with ASD (4.49–11.99 years) and 41 TD boys (4.75–12.16 years). IQ was characterized using the General Conceptual Ability score of the Differential Ability Scales-Second Edition (DAS-II) [57]. All participants were high-functioning (IQ > 70) (FSIQ_ASD_ = 78–136; FSIQ_TD_ = 78–140). As shown in Table 1, the ASD and TD groups were well-matched on age and IQ.

**Table 1:** Participant demographics and group matching

			Dx		Age × Dx	
Variable	TD (*n* = 41)	ASD (*n* = 60)	*t*_(99)_	*p*	*F*_(1,97)_	*p*
Age	8.83 (2.30)	8.35 (2.07)	1.09	0.28	−	−
General Conceptual Ability IQ	107.00 (15.02)	103.12 (14.51)	1.30	0.20	0.33	0.57
Verbal IQ	107.93 (12.20)	103.77 (17.18)	1.34	0.19	0.23	0.63
Non-verbal IQ	105.37 (15.33)	102.35 (13.82)	1.03	0.31	0.31	0.58

All participants with ASD met DSM-IV [58] diagnostic criteria for Asperger’s syndrome, pervasive developmental disorder—not otherwise specified, or autistic disorder as determined by expert clinical judgment. This judgment was supported by the results of gold standard diagnostic instruments, ADI-R [59] and/or ADOS [60], administered by research-reliable and licensed clinical psychologists. Autistic traits were measured using the parent-reported Social Responsiveness Scale (SRS) total raw score [61]. The complete characterization of the ASD group is reported in Table 2.

**Table 2:** ASD group characteristics

Variable	Mean (SD)
ADI-R (*n* = 56)	
Social	20.50 (5.86)
Verbal communication	16.55 (4.59)
Repetitive	5.95 (2.32)
ADOS Module 2 (*n* = 2)	
Social affect	13.00 (2.83)
Repetitive behaviors	5.00 (0.00)
Total	18.00 (2.83)
ADOS Module 3 (*n* = 58)	
Social affect	10.07 (3.47)
Repetitive behaviors	2.74 (1.74)
Total	12.81 (4.12)
ADOS Calibrated Severity score (*n* = 60)	7.43 (1.74)
SRS-parent total raw score (*n* = 58)	94.98 (30.30)

To rule out possible developmental delays/disorders and the Broad Autism Phenotype (BAP) [62, 63] in the TD participants, the following exclusionary criteria were used: (1) diagnosed or suspected ASD, schizophrenia, other developmental or psychiatric/neurological disorder; (2) first- or second-degree relative with diagnosed or suspected ASD; (3) an Individualized Education Program for special education services, including speech/language therapy, occupational therapy, and/or social skills intervention; (4) SRS-parent total *t*-score ≥ 76 (severe range); or (5) clinical impression of ASD, other developmental delay/disorder, BAP, or psychiatric disorder.

Exclusion criteria for all participants included seizures (owing to safety concerns during MRI scans), and a history of serious head injury or loss of consciousness. All participants passed MRI safety screening, including being free of any metal implants and evidence of claustrophobia. Written informed consent was obtained from each participant’s parent(s), and verbal assent was obtained from each participant. The Human Investigations Committee at Yale University approved this study.

### Structural imaging parameters

A high-resolution, T1-weighted, structural imaging sequence was obtained on a 3-Tesla Siemens Tim Trio scanner using a 32-channel head coil and MPRAGE pulse sequence (160 sagittal slices; slice thickness = 1 mm; repetition time = 1900 ms; echo time = 2.96 ms; number of excitations = 1; flip angle = 9°; inversion time = 900 ms; field of view = 256^2^ mm^2^; matrix = 256^2^; voxel size = 1.0 mm^3^).

### Cortical reconstruction

FreeSurfer image analysis suite version 5.1.0 (http://surfer.nmr.mgh.harvard.edu/) was employed to perform semi-automated cortical reconstruction. The technical details of these procedures are well-documented in prior publications [64, 65]. In one of the first stages, FreeSurfer registers the individual scans to the MNI305 atlas [66]. Afterwards, FreeSurfer constructs the boundary between white matter and cortical gray matter (this boundary is called the white surface) and the boundary between the gray matter and cerebrospinal fluid/dura (this boundary is called the pial surface). After these 3D surfaces are constructed, CT is estimated as the shortest distance from the white surface to the pial surface at each surface vertex. SA is measured by assigning an area to each vertex equal to the average of its surrounding triangles on the white surface [67]. CV is measured by the amount of gray matter volume that lies between the white surface and the pial surface [68]. CG is measured by the amount of cortex buried within the sulcal folds as compared with the amount of cortex on the outer visible cortex. As computed in FreeSurfer with additional flags [39], CG is quantified by a local gyrification index (LGI) in a 3D space, which is an extension of the 2D gyrification index originally proposed by Ziilles et al. [69]. For example, a LGI of 2.7 means that there is 2.7 times more cortical surface invaginated within the sulci in the surrounding area than the amount of visible cortical surface, while a LGI of 1 means that the cortex is flat in the surrounding area [70]. Most of the reconstruction procedure is automated and has been validated against histological analysis [71] and manual measurement [72]. The structural measures are created using spatial intensity gradients across tissue classes (not restricted to the voxel resolution of the original data) and are capable of detecting sub-millimeter differences.

### Quality assurance

In order to prevent any biases or confounds due to FreeSurfer version (workstation type or operating system version) [73], all reconstruction and segmentation computations were performed using computers of exactly the same workstation type and operating system (Dell PowerEdge M610 running CentOS 5.4) on the Yale High-Performance Computing cluster. Quality assurance of the reconstruction was achieved by visually inspecting the data from every participant. Two of the authors (DY and RJ) independently examined the structural MRI scans for ghosting and blurring. The inspection was performed with only the subject’s ID number, so that the raters were blind to information regarding diagnostic group, age, and IQ. Seven scans (6 ASD and 1 TD) were excluded because of severe ghosting/blurring due to head motion.

### Analytic approach

We performed group comparisons via GLM analyses using FreeSurfer in a two-step, step-down analytical approach. Age was demeaned in all analyses to ensure that results of the between-group difference refer to anatomy at mean age of the sample rather than age = 0 years. In the first step, we tested an age-by-diagnosis linear age interaction model using the DODS (“different offset, different slope”) method and the following GLM equation at every vertex: measure = *b*_**0**_ + *b*_**1**_ × age + *b*_**2**_ × diagnosis + *b*_**3**_ × age × diagnosis + error. The goal of the first step is to identify clusters of significant age-by-diagnosis interaction (that is, *b*_3_). In the second step, we screened out the clusters identified in the first step (if any) and tested everywhere else in the cortex using a simpler, between-group main effect model (that is, without the interaction term), the DOSS (“different offset, same slope”) method, and the following GLM equation: measure = *b*_**0**_ + *b*_**1**_ × age + *b*_**2**_ × diagnosis + error. The goal of the second step is to identify clusters of significant between-group difference independent of age (that is, *b*_2_). This step-down approach ensures that the clusters of significant between-group difference would not confound age-by-diagnosis interaction and thus increases the interpretability of the results. The DOSS method was used to ensure that no interaction term was erroneously included in the second step. The dependent variables were the four cortical structural measures: CT, SA, CV, and CG, respectively. For CT, SA, and CV, a smoothing kernel of 15-mm FWHM was implemented. For CG, because the LGI as implemented in FreeSurfer is already smooth by default, no smoothing kernel (FWHM = 0 mm) was implemented. All analyses were performed on each hemisphere separately. To correct for multiple comparisons and identify significant clusters, a cluster analysis (Monte Carlo null-Z simulation) [74] was implemented at a threshold of *p* < 0.05, two-sided. The statistics for the surviving clusters were reported (e.g., size of the cluster, cluster *p* value, peak vertex *t*-statistic, peak vertex coordinates in the Talairach space), while scatterplots (for age-by-diagnosis interaction effects) or boxplots (for diagnosis main effects) were generated to facilitate interpretation of the effects.

Within the ASD group, we performed similar two-step, step-down GLM analyses with the SRS total raw score as a continuous measure of autistic traits. Age and SRS total raw scores were demeaned in all analyses. In the first step, we tested an age-by-SRS total raw score linear age interaction model using the DODS (“different offset, different slope”) method and the following GLM equation at every vertex: measure = *b*_**0**_ + *b*_**1**_ × age + *b*_**2**_ × SRS + *b*_**3**_ × age × SRS + error. The goal of the first step is to identify clusters of significant age-by-SRS interaction (that is, *b*_3_). In the second step, we screened out the clusters identified in the first step (if any) and tested everywhere else in the cortex using a simpler, main effect model (that is, without the interaction term), the DOSS method, and the following GLM equation: measure = *b*_**0**_ + *b*_**1**_ × age + *b*_**2**_ × SRS + error. The goal of the second step is to identify clusters of a significant main effect of SRS total raw score that is independent of age (that is, *b*_2_). All the other procedures were the same as those used in the group comparison analyses.

To assess the degree to which there is a group difference on global brain anatomy (e.g., [13, 75]), we compared the two groups on several global measures, including total cortical gray matter volume, total cortical white matter volume, subcortical gray matter volume, total gray matter volume, and intra-cranial volume. As seen in Table 3, the two groups of participants were matched for all of these measures, *p*s > 0.72. This finding of global brain anatomy is similar to that from a previous study including preschool-aged children [33]. Furthermore, there was no significant age-by-diagnosis interaction on these global measures, *p*s > 0.05. That is, the two groups exhibited statistically comparable trajectories of global brain development. This is also the case for IQ (see Table 1). Together, these findings suggest that controlling for global brain measures or IQ in our data is not warranted and would also reduce power. Thus, we conducted the GLM analyses without controlling for global brain anatomy and IQ.

**Table 3:** Comparison of global brain anatomy between TD and ASD

			Dx		Age × Dx	
Global measure	TD	ASD	*t*_(99)_	*p*	*F*_(1,97)_	*p*
Cortical gray matter volume	477.79 (53.40)	476.49 (63.94)	0.11	0.92	3.38	0.07
Cortical white matter volume	437.69 (62.19)	434.02 (53.59)	0.32	0.75	0.12	0.73
Subcortical gray matter volume	194.13 (17.27)	193.18 (15.96)	0.29	0.78	0.01	0.92
Total gray matter volume	671.92 (64.18)	669.67 (71.47)	0.16	0.87	2.53	0.12
Intra-cranial volume	1318.38 (157.41)	1328.06 (108.79)	−0.37	0.72	0.11	0.74

The unit is cubic centimeters

### Meta-analytical reverse inference

To understand the functional relevance of the surviving clusters, we performed a quantitative reverse inference using Neurosynth (http://www.neurosynth.org/). At the time of this research, the Neurosynth dataset v0.5 contains activation data for over 10,900 studies and feature information for over 3300 term-based features. The term-based features were derived from the abstracts of articles in the Neurosynth database. For each feature, the database stores the whole-brain, reverse inference, meta-analysis map, *P*(Term|Activation), that is, the likelihood that a feature term is used in a study given the presence of reported activation [76]. For each effect of interest (e.g., significant age-by-diagnosis interaction effect) and for each cortical measure (e.g., CT), we merged the surface label files generated by FreeSurfer across all surviving clusters and then converted them to whole-brain volumetric NIfTI files in the standard space (MNI152) via *mri_label2vol*, a tool provided in the FreeSurfer package. Each of these NIfTI files was then decoded with Neurosynth, which computed the voxel-wise Pearson correlation between the input image file and the meta-analytical image file associated with each of the 3300 feature terms. The top 10 functional terms (e.g., spatial, working memory) with the highest positive correlation were retained and reported, while we omitted non-functional terms, such as (but not limited to) those describing an anatomical region (e.g., dorsolateral prefrontal), a technique (e.g., positron emission), or being relatively generic (e.g., task, valid, viewed).

## Results

### Step 1: age-by-diagnosis interaction effects

**Fig. 1:**
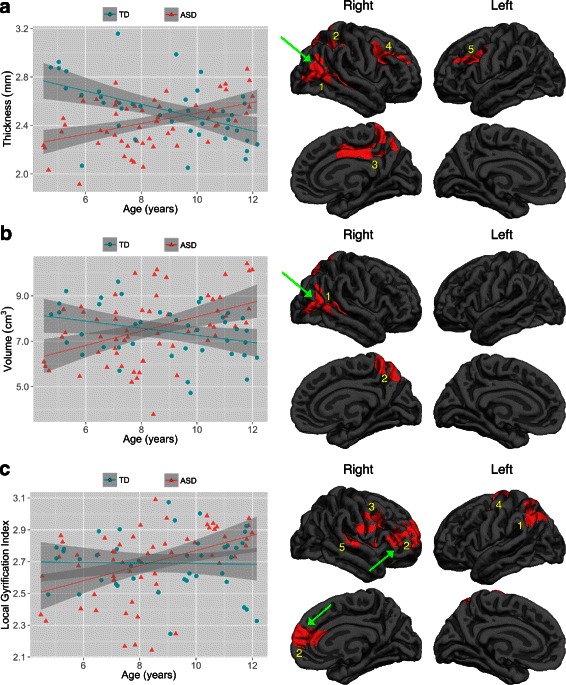
Clusters exhibiting significant age-by-diagnosis interaction effects on **a** cortical thickness, **b** cortical volume, and **c** cortical gyrification. The effects are illustrated by representative scatterplots. Results were corrected for multiple comparisons using cluster analysis, *p* < 0.05, two-sided. There were no surviving clusters for surface area. The numeric labels indicate distinct clusters, and the corresponding information associated with each cluster can be found in the tables. *Dark gray* = sulci; *light gray* = gyri

The first step of the two-step, step-down GLM analyses revealed several clusters of significant age-by-diagnosis interaction effects on CT, CV, and CG but not SA. As illustrated by representative scatterplots in Fig. 1, on CT, ASD relative to TD exhibited a lack of normative age-related cortical thinning during childhood in several regions, with the peaks found in the right middle temporal, superior parietal, posterior cingulate, rostral middle frontal, and the left caudal middle frontal gyri. On SA, ASD and TD exhibited statistically comparable developmental trajectories. On CV, ASD relative to TD exhibited a lack of normative age-related reduction during childhood in gray matter volume in several regions, with the peaks found in the right banks of the superior temporal sulcus and the right superior parietal gyrus. Finally, on CG, ASD relative to TD tended to have abnormal age-related increase during childhood in several regions, with the peaks found in the left superior parietal, precentral, right rostral middle frontal, pars opercularis, and superior temporal gyri. Information about all clusters is reported in Table 4 (top), while comprehensive information concerning each cluster is reported in Additional file 1: Figures S1–S3 and Table S1.

**Table 4:** Clusters of significant differences in cortical morphometry between TD and ASD

Measure	Cluster	Size (mm^2^)	*N*_vertices_	*p*_cluster_	*t*_peak_	*X*_Tal_	*Y*_Tal_	*Z*_Tal_	Peak region
Age-by-diagnosis interaction effects (*df* = 97)									
Thickness	1	2965.01	5623	0.0002	4.334	50.3	−56.7	8.2	R middle temporal
	2	2864.20	6405	0.0002	3.867	19.0	−64.6	43.5	R superior parietal
	3	2558.92	6841	0.0005	4.743	14.6	−14.4	37.4	R posterior cingulate
	4	1878.90	3472	0.0080	3.750	26.5	36.7	18.1	R rostral middle frontal
	5	1457.79	2597	0.0375	3.885	−33.0	9.4	28.1	L caudal middle frontal
Volume	1	2370.44	4619	0.0002	4.677	48.1	−33.0	4.5	R bankssts
	2	1912.43	4245	0.0015	3.658	18.8	−64.9	43.6	R superior parietal
Gyrification	1	2662.70	6064	0.0001	3.361	−27.8	−49.8	48.5	L superior parietal
	2	4359.25	6920	0.0001	3.277	20.2	58.7	8.9	R rostral middle frontal
	3	2141.64	4528	0.0001	3.243	35.6	8.4	21.8	R pars opercularis
	4	995.32	2324	0.0013	3.586	−17.2	−13.4	57.9	L precentral
	5	824.99	1881	0.0067	3.047	64.9	−20.1	2.4	R superior temporal
Between-group differences (independent of Age) (*df* = 98)									
Gyrification	1	2275.23	3856	0.0001	3.862	38.9	−78.1	20.8	R inferior parietal
	2	1625.12	2784	0.0001	3.820	51.2	−13.5	−30.5	R inferior temporal
	3	4186.02	7911	0.0001	3.645	−13.5	−48.8	6.2	L isthmus cingulate
	4	1738.54	2122	0.0001	3.644	10.4	−91.7	−1.7	R lingual

### Step 2: diagnosis main effects (independent of age)

**Fig. 2:**
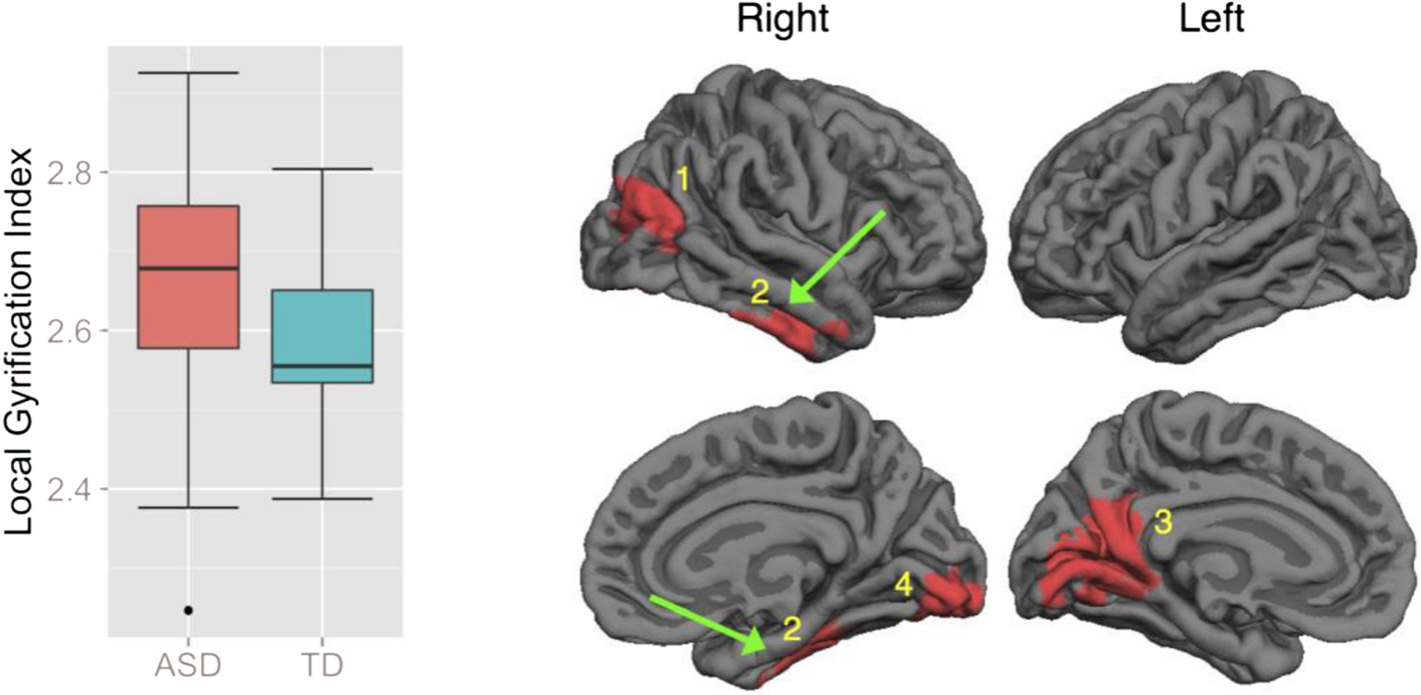
Clusters exhibiting significant between-group differences independent of age on cortical gyrification. The effects are illustrated by a representative boxplot. Results were corrected for multiple comparisons using cluster analysis, *p* < 0.05, two-sided. There were no surviving clusters for cortical thickness, surface area, and cortical volume. The numeric labels indicate distinct clusters, and the corresponding information associated with each cluster can be found in the tables. *Dark gray* = sulci; *light gray* = gyri

The second step of the GLM analyses revealed several clusters of significant between-group difference effects independent of age on CG but not CT, SA, and CV. As illustrated by a representative boxplot in Fig. 2, ASD relative to TD tended to have higher mean levels of gyrification in several regions when covarying for age across childhood. The peaks were localized to the right inferior parietal, inferior temporal, lingual, and the left isthmus cingulate gyri. Information about all clusters is reported in Table 4 (bottom), while comprehensive information concerning each cluster is reported in Additional file 1: Figure S4.

### Analyses of autistic traits within the ASD group

**Fig. 3:**
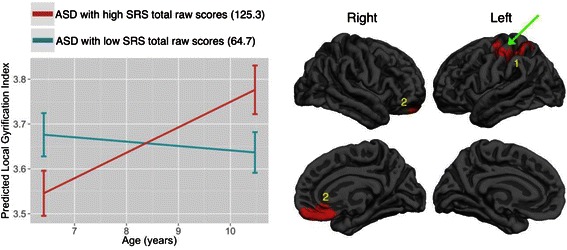
Clusters exhibiting significant age-by-SRS total raw scores interaction effects within the ASD group on cortical gyrification. The effects are illustrated by a representative interaction plot using predicted gyrification values conditional upon high and low levels of SRS total raw scores (*M* ± 1 SD) and high and low levels of age (*M* ± 1 SD). The *error bars* indicate standard errors of the mean. There were no surviving clusters for cortical thickness, surface area, and cortical volume. The numeric labels indicate distinct clusters, and the corresponding information associated with each cluster can be found in the tables. *Dark gray* = sulci; *light gray* = gyri

The first step of the two-step, step-down GLM analyses within the ASD group revealed two clusters of significant age-by-SRS total raw score interaction effects on CG but not CT, SA, or CV. As illustrated by a representative interaction plot in Fig. 3 using predicted gyrification values conditional upon high and low levels of SRS total raw scores (*M* ± 1 SD) and high and low levels of age (*M* ± 1 SD), those with higher SRS total raw scores (125.3) relative to those with lower SRS total raw scores (64.7) within the ASD group tended to have greater age-related increase in gyrification in specific regions during childhood. The peaks were found in the left precentral and the right medial orbitofrontal gyri. There were no surviving clusters from the second step of the GLM analyses. Information about both clusters is reported in Table 5, while comprehensive information concerning each cluster is reported in Additional file 1: Figure S5 and Table S2.

**Table 5:** Clusters of significant age-by-SRS total raw score interaction on cortical gyrification within ASD

Measure	Cluster	Size (mm^2^)	*N*_vertices_	*p*_cluster_	*t*_peak(54)_	*X*_Tal_	*Y*_Tal_	*Z*_Tal_	Peak region
Gyrification	1	2950.74	7125	0.0001	3.415	−28.5	−10.3	50.4	L precentral
	2	1319.11	2542	0.0001	2.803	7.6	53.5	−18.7	R medial orbitofrontal

## Discussion

The study sets out to examine the cortical morphological differences in a large sample of children with ASD, 4–12 years of age, across all four SBM measures: CT, SA, CV, and CG. Currently, there have been relatively few neuroanatomical studies examining children with ASD in this age range and even fewer studies included all of the four measures at the same time. Our results showed that while there was no age-by-group interaction or between-group difference on global brain anatomy, there were significant age-by-group interactions and between-group differences on regional brain anatomy in CT, CV, and CG but not SA. Our principal findings also held when global brain anatomy (e.g., intra-cranial volume) and IQ were included as covariates, either separately or together. To interpret the functional relevance of the surviving clusters, we conducted a Neurosynth-based quantitative reverse inference (see Table 6; image files available at http://neurovault.org/collections/1073/; interested readers may decode the image files with NeuroSynth through links within the NeuroVault website). Strikingly, many of the clusters are related to social perception, language, self-referential, and action observation networks—those frequently found to be functionally altered in individuals with ASD [6, 77, 78]. In brief, these neurodevelopmental structural differences and the use of multiple cortical measures may contribute to a more comprehensive picture of the underlying processes of the social communication difficulties that characterize children with ASD.

**Table 6:** Quantitative reverse inference of the surviving clusters using Neurosynth

Measure	Top 10 Neurosynth-decoded feature terms
Clusters of significant age-by-diagnosis interaction effects	
Thickness	Spatial (0.11), working memory (0.10), visuospatial (0.10), attentional (0.09), biological (0.08), motion (0.08), attention (0.07), action observation (0.07), moving (0.07), location (0.06)
Volume	Biological (0.15), motion (0.15), spatial (0.12), location (0.12), orienting (0.11), moving (0.11), visuospatial (0.11), perception (0.10), facial expression (0.09), gaze (0.09)
Gyrification	Pitch (0.09), working memory (0.09), production (0.08), sound (0.07), tone (0.07), vocal (0.07), executive function (0.07), speech production (0.07), audiovisual (0.07), noises (0.06)
Clusters of significant between-group differences (independent of age)	
Gyrification	Navigation (0.08), autobiographical memory (0.08), episodic (0.08), motion (0.07), memories (0.06), relational (0.06), semantic memory (0.06), self-referential (0.04), interpersonal (0.04), mental states (0.04)
Clusters of significant age-by-SRS total raw score interaction effects within ASD	
Gyrification	Hand (0.22), finger (0.22), grasping (0.22), movements (0.18), execution (0.17), action (0.15), motion imagery (0.10), reaching (0.10), touch (0.08), action observation (0.07)

The numbers within the parentheses are correlation coefficients between the surviving clusters and the meta-analysis maps of the feature terms in Neurosynth

### Age-by-diagnosis interaction effects

The results showed that ASD relative to TD showed a lack of normative age-related reduction in CT [79] and CV in specific regions during childhood. A closer look at the regions (see Additional file 1: Table S1) reveals that ASD also had abnormal, age-related expansion in CT and CV in specific regions, particularly near the right middle temporal gyrus and the posterior superior temporal sulcus. Recently, there were two longitudinal studies that showed an increase in age-related reduction in CT in ASD during adolescence [21, 80] and on the surface, their findings may appear to be at odds with ours. The discrepancies between these works and ours are summarized here. In the study of Zielinski et al. (2014) [21], the sample consisted of not just children but also many adolescents and adults (age range was 3–36 years; mean age at scan 1 was 15 years) and quadratic age effects were modeled. Thus, their findings during childhood likely reflect a portion of the quadratic age effect across a much wider age range and can have been heavily influenced by the non-linear development in adolescence and then adulthood. Statistically speaking, the quadratic age effect allows only one turning point in the developmental trajectory, which appears to be around late adolescence and early adulthood in their study. We suspect that if a cubic age effect had been modeled, which statistically allows two turning points in the developmental trajectory (e.g., around early adolescence and around early adulthood, respectively [81]), the age effect during childhood in this previous study might have been more similar to ours. Similarly, a recent volumetric analysis including individuals with ASD 3–35 years of age [45] found accelerated decreases in volumes in ASD with age and suggested that the quadratic age effects are primarily due to decreases during late adolescence and adulthood. In contrast, our model fits just the children data using a linear age effect model and captures the childhood phenomena via a different developmental window and statistical model. In addition, in both studies of Wallace et al. (2015) [80] and Zielinski et al. (2014) [21], the main finding is that there is an increase in age-related cortical thinning in ASD during adolescence. This finding is not necessarily in conflict with our finding. Coupled with our finding, the overall picture suggests that abnormal cortical thickening and a lack of age-related normative cortical thinning in ASD during childhood (our finding) is unlikely to continue indefinitely and may be followed by a rebound effect of increased cortical thinning in ASD during adolescence (the findings in both previous studies). In brief, the findings that seem to be in conflict on the surface may have deeper meaningful connections that can prompt new investigations. Future studies should collect a longitudinal sample with more children, a wider age range, and more data time points to further examine this possibility.

In contrast, there was no group difference in the linear developmental trajectory in SA. Despite a null finding, it is consistent with previous research that included adolescents [56] and adolescents and adults [80]. Currently, there is a relative dearth of studies that examine SA. Among them, the significant findings have been mixed and inconsistent. For example, studies that used younger subjects (e.g., aged 0–5 years) found that at an early age, the ASD group has increased SA [13, 82], a study that used adult subjects showed reduced SA [83], but a recent longitudinal study that included children, adolescents, and adults found that the ASD group relative to controls had reduced SA during childhood and increased SA during adulthood in specific regions [84]. Clearly, more work is needed here to unveil the dynamic developmental patterns of surface area in ASD relative to normative developmental patterns.

On CG, the ASD group relative to TD showed an abnormal age-related increase of cortical folding in a number of regions in the right prefrontal lobe, the right temporal lobe, and the left parietal lobe. Within the ASD group, those with higher autistic traits also exhibited age-related CG increase in the right prefrontal lobe and the left parietal lobe. There are currently few studies that have examined developmental trajectories in CG in ASD. More work is also needed here to understand the CG developmental patterns in ASD, relative to normative patterns. Importantly, CG is thought to be associated with local neural connectivity in childhood and adolescence [48]. Our finding of accelerated expansion of CG in the frontal cortex is thus in line with a view that there is local over-connectivity in the frontal cortex in ASD [85]. Furthermore, previous research that included older subjects (adolescents and adults) demonstrated an age-related decrease of cortical folding in ASD but no change in TD [54]. Together with our results, these results suggest that CG in TD is largely a constant from childhood to adulthood [48, 86], while ASD may have abnormal, age-related CG increase during childhood, which is followed by abnormal, age-related CG decline during adolescence and adulthood. In sum, the ASD group relative to TD showed altered developmental trajectories in CT, CV, and CG, suggesting age-related brain structural dysmaturation in childhood in ASD.

### Diagnosis main effects (independent of age)

Our results showed that the ASD group relative to TD had higher mean levels of CG independent of age in the temporal, parietal, and occipital lobes and part of the cingulate cortex, which is consistent with the previous findings of increased CG in ASD [54, 56]. Greater CG is found to be related to greater local connectivity [51], suggesting that the ASD group may have local hyper-connectivity in these regions [87]. It is important to note that our two-step, step-down approach helps to clarify whether the regions are about between-group difference or age-by-diagnosis interactions. This procedure ensures that the between-group difference regions are not moderated by age and can be clearly interpreted. The CG results are highly region-specific, that is, different regions showed age-by-diagnosis interactions or between-group differences. More work is needed to understand the nature of the specific regions of CG increase in ASD.

There were no surviving regions that showed between-group difference in CT, SA, or CV. In light of the age-by-diagnosis findings, our results suggest that the CT and CV cortical differences in ASD during childhood are primarily dynamic, age-dependent, and unfolded in the developmental trajectories over time. A previous similar study by Raznahan et al. (2013) [33] found that children with ASD showed age-independent thicker cortices in specific regions across 2 to 5 years of age. In contrast, the current work included children with ASD 4 to 12 years of age but we did not identify any region that showed mean-level CT difference independent of age. It is possible that there is a cohort effect and also there may be an effect of a wider vs. narrower age range. That is, a wider age range might afford more of the opportunity to examine age-dependent dynamic differences, whereas a narrower age range might inherently limit the results to the static, age-independent, mean-level differences. Future work should include a sample of a wider age range to further understand the discrepancies in these findings.

### The importance of using multiple cortical measures

Using all four key cortical measures in a surface-based morphometry study, our results demonstrate the importance of understanding the neuroanatomical basis of ASD using multiple cortical measures. These four measures likely have distinct genetic, environmental, cellular, and biomechanic determinants. Our finding of the ASD difference in the linear developmental trajectories in CV tends to be more similar to that in CT than in SA, suggesting that the CV developmental difference in ASD during childhood may be largely attributable to CT, rather than SA. In light of the radial unit hypothesis [41], our CT findings suggest that individuals with ASD may fail to have normative age-related reduction in the number or even size of the neuronal cell bodies within the cortical minicolumns in specific cortical regions during childhood. In contrast, our SA finding does not support that individuals with ASD may have abnormal proliferation or decline in the numbers of cortical minicolumns during childhood.

Our CG findings are similar to the CT and CV findings in that ASD participants generally exhibited abnormal age-related increase across these cortical matrices during childhood. However, the CG findings also provide unique information about ASD abnormality. In our results, CG is the only measure that not only showed ASD vs. TD difference in neurodevelopmental trajectory but also captured ASD vs. TD difference in the age-independent mean neuroanatomical difference across childhood and the age-related neuroanatomical abnormality associated with higher levels of autistic traits within the ASD group. This implies that CG may serve as a highly sensitive indicator of ASD abnormality across multiple levels of analysis. Nonetheless, CG differences were seen in a different set of cortical regions (vs. CT and CV) and CG does not replace the roles of CT and CG measures. Currently, there are scant CG works in the literature and more CG work should been done in the future in order to more fully understand the meaning and the underlying cause of the ASD difference in this cortical measure. In summary, it is highly beneficial and important to understand ASD via all four cortical measures, which help to chart a fuller picture of the complexity of ASD and may generate novel and useful hypotheses and research directions.

### Limitations

Several limitations of this study are important to consider. First, quality assurance relied on careful visual inspection and data elimination. However, it is likely that individuals with ASD who exhibit more severe symptoms would also find it harder to remain still in the MRI scanner for a long period of time and thus there may be a selection bias in our data. Future research should employ real-time prospective motion correction (PMC) techniques (e.g., [88–90]) during the sMRI scan. The use of PMC techniques can also prevent the risk of using sedation, is more likely to be approved by most IRB for research purpose, and may be a better option than sedation for future sMRI research that aims at minimizing head motion confounds [91]. Second, our data are cross-sectional in nature and the results from the age-related analyses were based on different individuals. Consequently, our results could not rule out the alternative interpretation that the findings reflect cohort differences, rather than true developmental trajectories, although our TD results of cortical thinning with age are highly congruent with established findings [79]. Future studies should use longitudinal data to further establish the findings. Third, our sample consisted of predominantly high-functioning children with ASD and it remains unclear whether the findings can generalize to children with ASD who show mild to severe intellectual disability. To address this issue, future studies should include individuals with intellectual disability in both the ASD and control groups. Fourth, this study only examined linear age effects and interactions. However, key cortical measures such as CT have been shown to develop in a highly non-linear fashion [81] and recent research has begun to unveil quadratic age-related neurodevelopmental difference in CT in ASD [21, 84]. To properly test the quadratic and also cubic age effects, it would require a sample of a much wider age range than the current sample (e.g., three age cohorts: 4–12, 12–18, and 18–25 years of age, and a large enough number of participants in each age cohort). A minimum of four time points in a longitudinal study has been suggested to be required for reliable quadratic trajectories at the individual level with a cohort sequential sampling design [92]. In contrast, the current sample included exclusively children 4–12 years of age. While it provides a developmental window to test the linear age effect, our sample is not well-suited to carry out the non-linear age-related statistical tests. Future studies should collect a much larger sample of a wider age range to test these more complicated age effects. Finally, the current study only included boys. While males with ASD outnumber females with ASD in the population, our results may be only applicable to male children with ASD because there is a sex difference in the brain structural development [93]. It is important for future studies to collect structural MRI data on female children with ASD to characterize any gender-specific neuroanatomical differences in children with ASD.

## Conclusions

The current study is the first to examine cortical thickness, surface area, cortical volume, and cortical gyrification in the same study that included pre-adolescent children with ASD. In addition to rigorous characterization in ASD participants, this study includes a TD comparison group that was well-matched on sex, age, and IQ, which help to minimize possible confounds. The four cortical morphological measures describe a complex but rich picture of the neurodevelopmental structural differences in ASD in this age range. Our sample sizes are relatively large, with 60 participants in ASD and 41 participants in TD, which increase the reliability of the results and the statistical power to detect true effects in MRI studies, especially in heterogeneous populations such as ASD [94]. Given the known challenges of heterogeneity in the ASD population, future studies should continue to recruit large-scale samples to ensure greater reliability and reproducibility of results. Moreover, being a lifetime neurodevelopmental disorder, future studies should collect longitudinal data at different time points across the lifespan from infancy to late adulthood and apply the approach of multiple morphological measures to help depict a more comprehensive picture of ASD.

## Additional file

Additional file 1:
**Tables S1 and S2, Figures S1–S5. Table S1.** Age-related regression effects by diagnosis on cortical measures. **Table S2.** Age-related regression effects conditional upon high and low levels of SRS total raw scores within ASD on cortical gyrification. **Figures S1–S3.** Clusters exhibiting significant age-by-diagnosis interaction effects on (S1) cortical thickness, (S2) cortical volume, and (S3) cortical gyrification. The effects are illustrated by corresponding scatterplots. Results were corrected for multiple comparisons using cluster analysis, *p* < 0.05, two-sided. There were no surviving clusters for surface area. The numeric labels indicate distinct clusters and the corresponding information associated with each cluster can be found in the tables. Dark gray = sulci; light gray = gyri. **Figure S4.** Clusters exhibiting significant between-group differences independent of age on cortical gyrification. The effects are illustrated by corresponding boxplots. Results were corrected for multiple comparisons using cluster analysis, *p* < 0.05, two-sided. There were no surviving clusters for cortical thickness, surface area, and cortical volume. The numeric labels indicate distinct clusters and the corresponding information associated with each cluster can be found in the tables. Dark gray = sulci; light gray = gyri. **Figure S5.** Clusters exhibiting significant age-by-SRS total raw scores interaction effects within the ASD group on cortical gyrification. The effects are illustrated by corresponding interaction plots using predicted gyrification values conditional upon high and low levels of SRS total raw scores (*M* ± 1 SD) and high and low levels of age (*M* ± 1 SD). The error bars indicate standard errors of the mean. There were no surviving clusters for cortical thickness, surface area, and cortical volume. The numeric labels indicate distinct clusters and the corresponding information associated with each cluster can be found in the tables. Dark gray = sulci; light gray = gyri. (DOCX 2341 kb)

## Abbreviations

ADI-R: The Autism Diagnostic Interview Revised
ADOS: The Autism Diagnostic Observation Schedule
ASD: autism spectrum disorder
CG: cortical gyrification
CSS: calibrated severity score
CT: cortical thickness
CV: cortical volume
fMRI: functional magnetic resonance imaging
IPC: intermediate progenitor cell
LGI: local gyrification index
NIfTI: Neuroimaging Informatics Technology Initiative
PMC: prospective motion correction
SA: surface area
SBM: surface-based morphometry
sMRI: structural magnetic resonance imaging
SRS: Social Responsiveness Scale
STS: superior temporal sulcus
TD: typically developing
VBM: voxel-based morphometry
