# Supplementary material for: Cortical morphological markers in children with autism: a structural magnetic resonance imaging study of thickness, area, volume, and gyrification
Source: Mol Autism. 2016 Jan 25;7:11. doi: 10.1186/s13229-016-0076-x (PMC4727390; doi:10.1186/s13229-016-0076-x)

**Additional file 1**

**Table S1** Age-related regression effects by diagnosis on cortical measures

|  |  | TD | | | | |  | ASD | | | | |
| --- | --- | --- | --- | --- | --- | --- | --- | --- | --- | --- | --- | --- |
| Measure | Cluster | *b* | β | *t*_(39)_ | *p* | *sig.* |  | *b* | β | *t*_(58)_ | *p* | *sig.* |
| Clusters of significant Age-by-Diagnosis interaction effects | | | | | | | | | | | | |
| Thickness | 1 | -.0550 | -.51 | -3.67 | .0007 | *** |  | .0436 | .45 | 3.82 | .0003 | *** |
|  | 2 | -.0772 | -.66 | -5.42 | < .0001 | *** |  | .0244 | .25 | 1.98 | .0520 |  |
|  | 3 | -.0731 | -.73 | -6.69 | < .0001 | *** |  | .0219 | .23 | 1.83 | .0729 |  |
|  | 4 | -.0658 | -.63 | -5.10 | < .0001 | *** |  | .0251 | .25 | 2.01 | .0497 | * |
|  | 5 | -.0603 | -.65 | -5.37 | < .0001 | *** |  | .0243 | .26 | 2.01 | .0489 | * |
| Volume | 1 | -.1565 | -.31 | -2.06 | .0462 | * |  | .3155 | .44 | 3.70 | .0005 | *** |
|  | 2 | -.2815 | -.53 | -3.95 | .0003 | *** |  | .0828 | .16 | 1.25 | .2180 |  |
| Gyrification | 1 | -.0250 | -.26 | -1.71 | .0955 |  |  | .0254 | .28 | 2.21 | .0311 | * |
|  | 2 | -.0019 | -.03 | -0.15 | .8780 |  |  | .0476 | .44 | 3.72 | .0004 | *** |
|  | 3 | -.0129 | -.09 | -0.59 | .5560 |  |  | .0609 | .39 | 3.21 | .0021 | ** |
|  | 4 | -.0350 | -.39 | -2.64 | .0119 | * |  | .0265 | .28 | 2.20 | .0318 | * |
|  | 5 | .0008 | .01 | 0.03 | .9750 |  |  | .0842 | .41 | 3.46 | .0010 | ** |
| Clusters of significant between-group differences (independent of Age) | | | | | | | | | | | | |
| Gyrification | 1 | -.0194 | -.24 | -1.54 | .1320 |  |  | -.0134 | -.14 | -1.04 | .3030 |  |
|  | 2 | -.0053 | -.13 | -0.79 | .4330 |  |  | -.0088 | -.15 | -1.15 | .2560 |  |
|  | 3 | -.0148 | -.18 | -1.15 | .2580 |  |  | .0009 | .01 | 0.07 | .9490 |  |
|  | 4 | -.0080 | -.16 | -0.99 | .3290 |  |  | -.0108 | -.16 | -1.21 | .2320 |  |

*Note*. *b* (β) refers to unstandardized (standardized) regression coefficients.

**p* < .05 ***p* < .01 ****p* < .001

**Table S2** Age-related regression effects conditional upon high and low levels of SRS total raw scores within ASD on cortical gyrification

|  |  | Low SRS total raw scores (64.7) | | | | |  | High SRS total raw scores (125.3) | | | | |
| --- | --- | --- | --- | --- | --- | --- | --- | --- | --- | --- | --- | --- |
| Measure | Cluster | *b* | β | *t*_(58)_ | *p* | *sig.* |  | *b* | β | *t*_(58)_ | *p* | *sig.* |
| Gyrification | 1 | -.0097 | -.10 | -0.62 | .5390 |  |  | .0567 | .60 | 2.92 | .0051 | ** |
|  | 2 | -.0018 | -.03 | -0.19 | .8520 |  |  | .0378 | .66 | 3.24 | .0020 | ** |

*Note*. *b* (β) refers to unstandardized (standardized) regression coefficients.

***p* < .01

**Figure Captions**

***Figures S1-S3.*** Clusters exhibiting significant age-by-diagnosis interaction effects on (**S1**) cortical thickness, (**S2**) cortical volume, and (**S3**) cortical gyrification. The effects are illustrated by corresponding scatterplots. Results were corrected for multiple comparisons using cluster analysis, *p* < .05, two-sided. There were no surviving clusters for surface area. The numeric labels indicate distinct clusters and the corresponding information associated with each cluster can be found in the tables. Dark gray = sulci; light gray = gyri.

***Figure S4.*** Clusters exhibiting significant between-group differences independent of age on cortical gyrification. The effects are illustrated by corresponding boxplots. Results were corrected for multiple comparisons using cluster analysis, *p* < .05, two-sided. There were no surviving clusters for cortical thickness, surface area, and cortical volume. The numeric labels indicate distinct clusters and the corresponding information associated with each cluster can be found in the tables. Dark gray = sulci; light gray = gyri.

***Figure S5*.** Clusters exhibiting significant age-by-SRS total raw scores interaction effects within the ASD group on cortical gyrification. The effects are illustrated by corresponding interaction plots using predicted gyrification values conditional upon high and low levels of SRS total raw scores (*M* ± 1*SD*) and high and low levels of Age (*M* ± 1*SD*). The error bars indicate standard errors of the mean. There were no surviving clusters for cortical thickness, surface area, and cortical volume. The numeric labels indicate distinct clusters and the corresponding information associated with each cluster can be found in the tables. Dark gray = sulci; light gray = gyri.

**Figure S1**


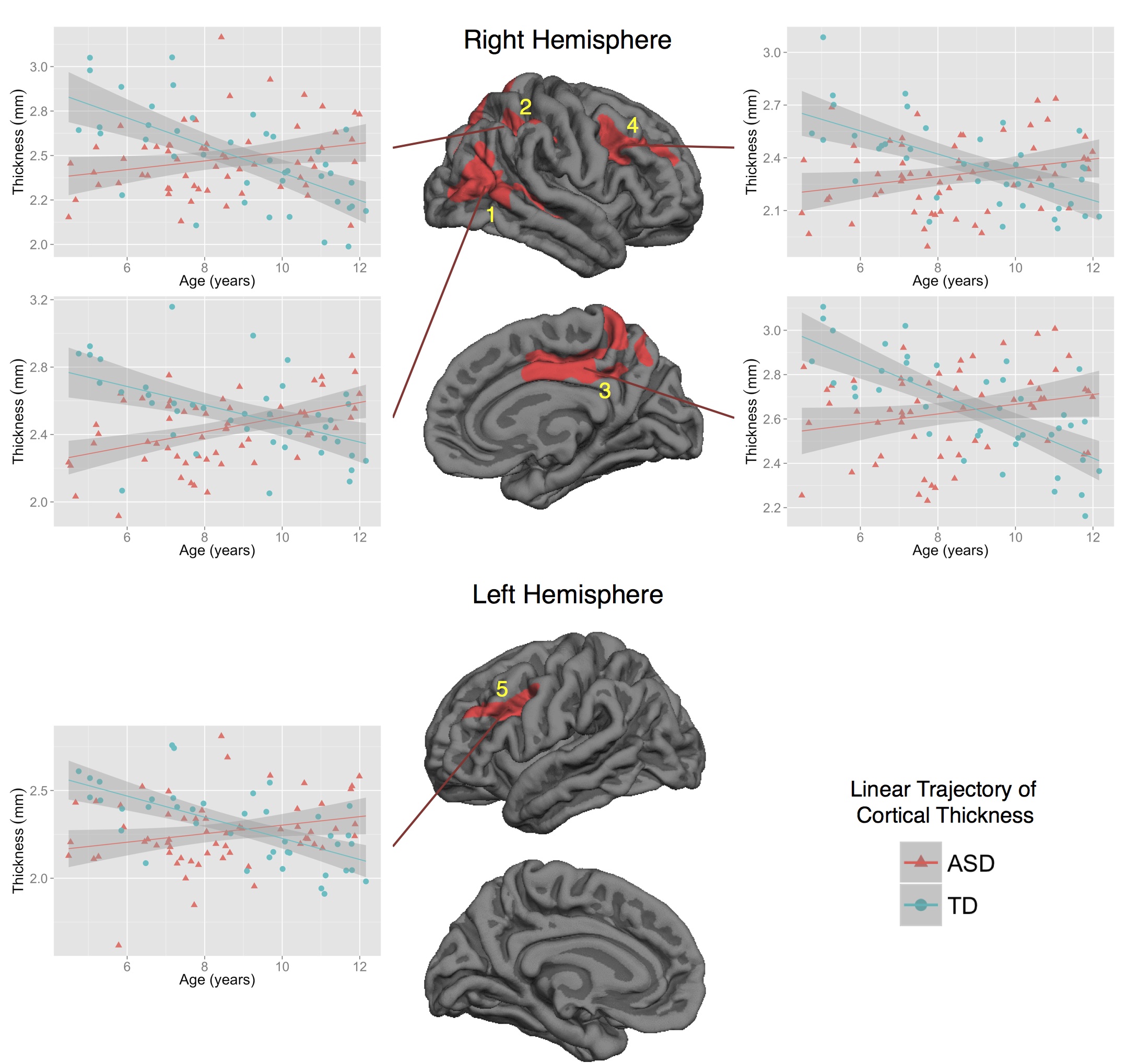


**Figure S2**


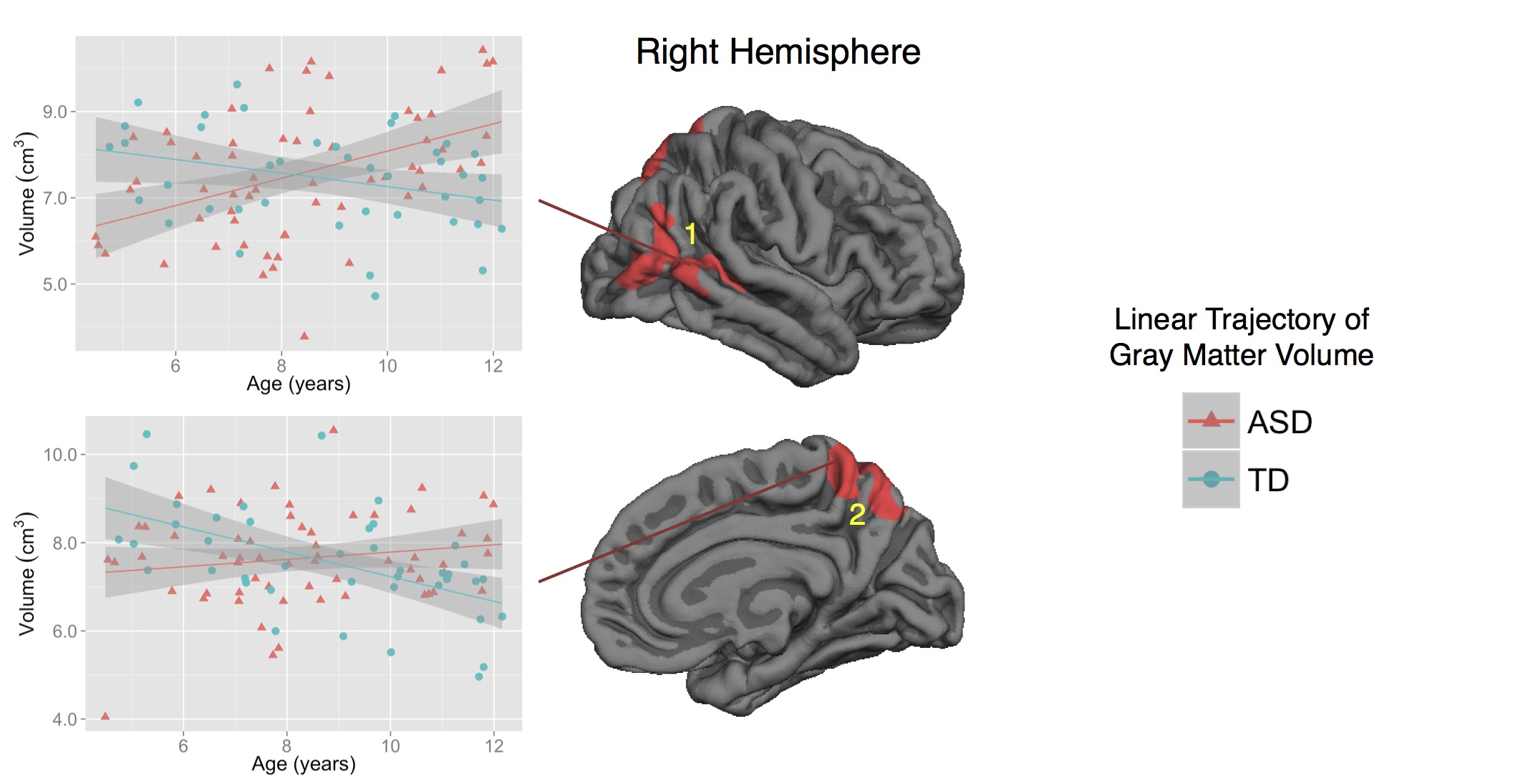


**Figure S3**


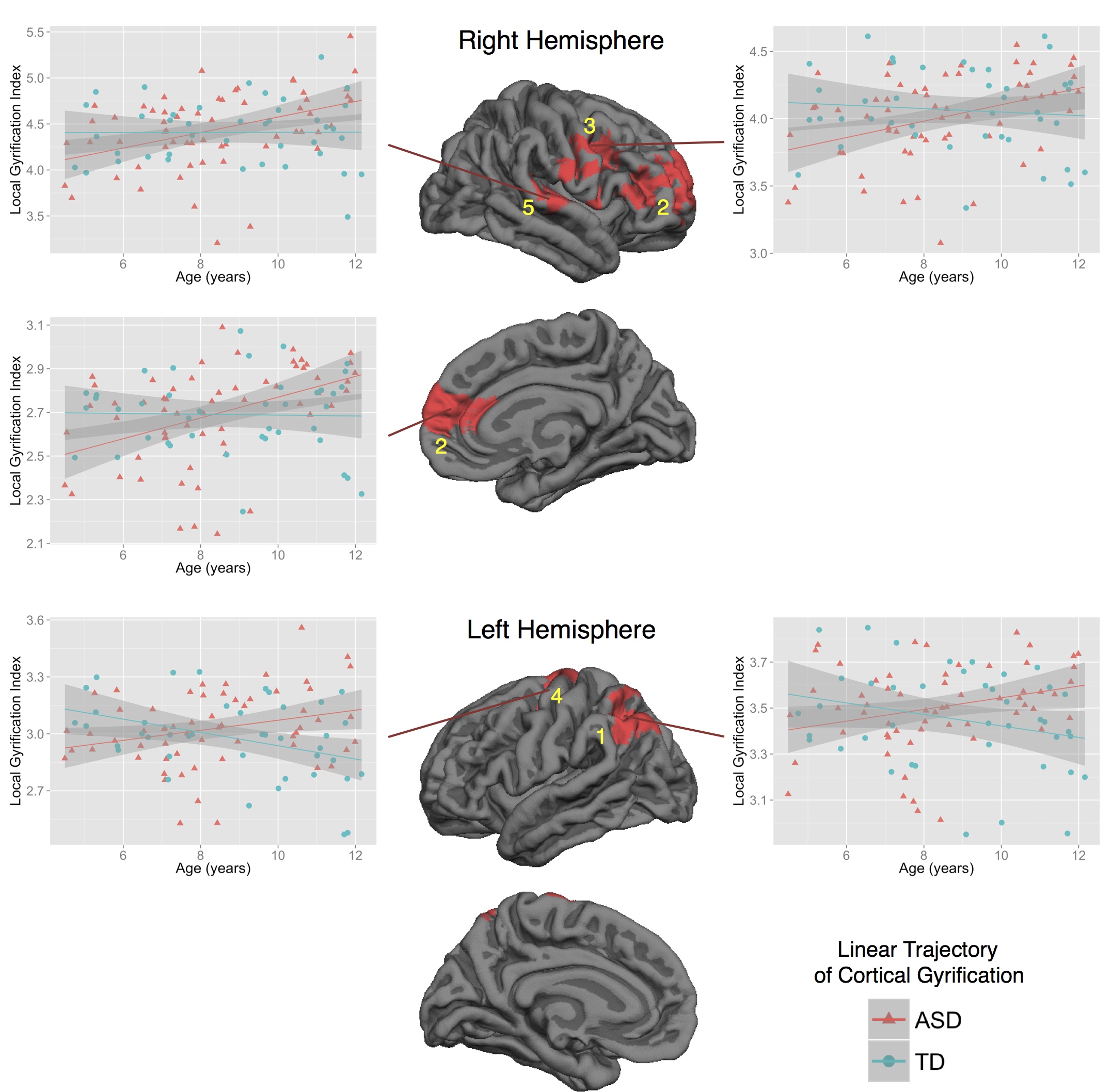


**Figure S4**


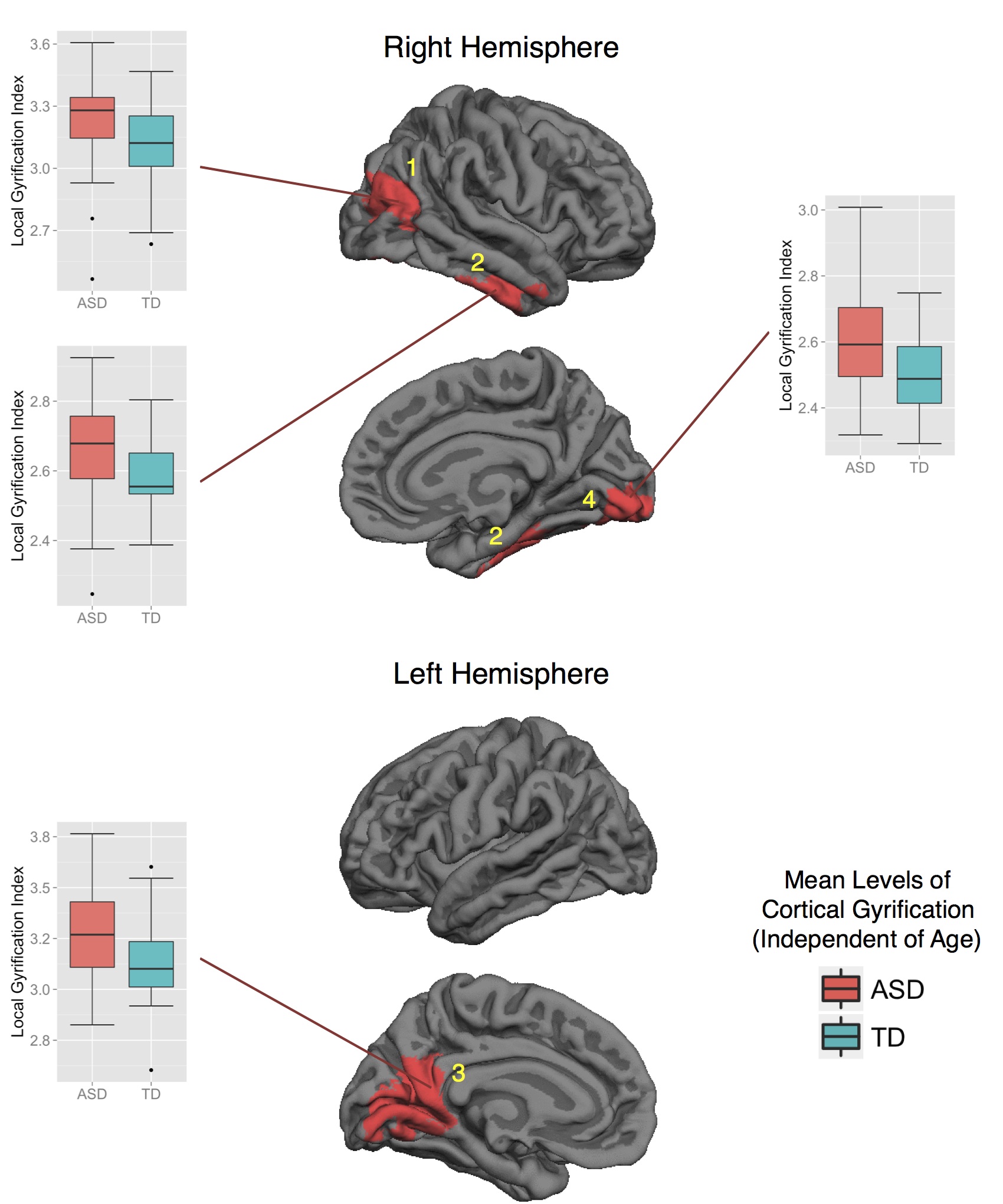


**Figure S5**


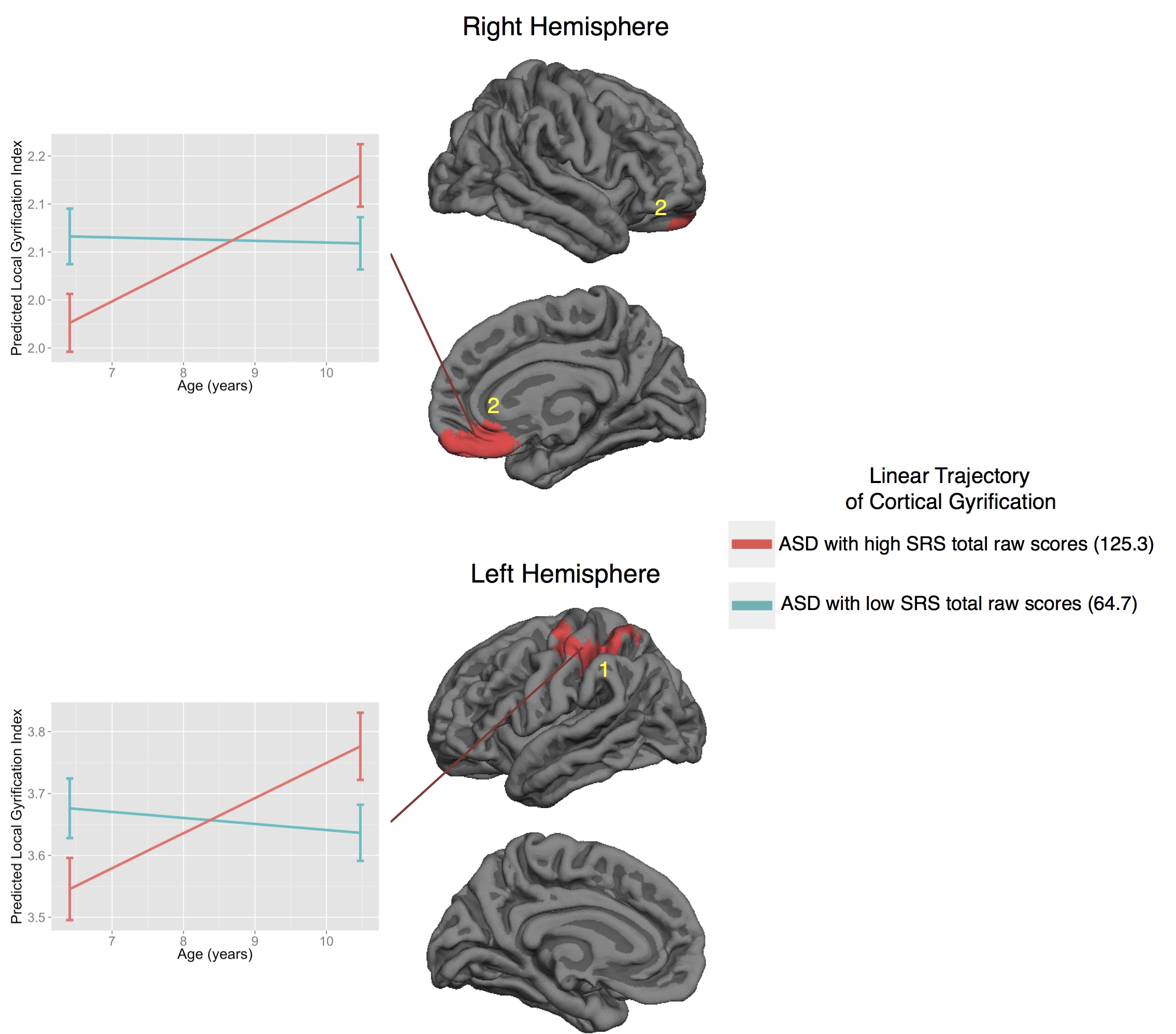

Supplement: Additional file 1: — Tables S1 and S2, Figures S1–S5. Table S1. Age-related regression effects by diagnosis on cortical measures. Table S2. Age-related regression effects conditional upon high and low levels of SRS total raw scores within ASD on cortical gyrification. Figures S1–S3. Clusters exhibiting significant age-by-diagnosis interaction effects on (S1) cortical thickness, (S2) cortical volume, and (S3) cortical gyrification. The effects are illustrated by corresponding scatterplots. Results were corrected for multiple comparisons using cluster analysis, p < 0.05, two-sided. There were no surviving clusters for surface area. The numeric labels indicate distinct clusters and the corresponding information associated with each cluster can be found in the tables. Dark gray = sulci; light gray = gyri. Figure S4. Clusters exhibiting significant between-group differences independent of age on cortical gyrification. The effects are illustrated by corresponding boxplots. Results were corrected for multiple comparisons using cluster analysis, p < 0.05, two-sided. There were no surviving clusters for cortical thickness, surface area, and cortical volume. The numeric labels indicate distinct clusters and the corresponding information associated with each cluster can be found in the tables. Dark gray = sulci; light gray = gyri. Figure S5. Clusters exhibiting significant age-by-SRS total raw scores interaction effects within the ASD group on cortical gyrification. The effects are illustrated by corresponding interaction plots using predicted gyrification values conditional upon high and low levels of SRS total raw scores (M ± 1 SD) and high and low levels of age (M ± 1 SD). The error bars indicate standard errors of the mean. There were no surviving clusters for cortical thickness, surface area, and cortical volume. The numeric labels indicate distinct clusters and the corresponding information associated with each cluster can be found in the tables. Dark gray = sulci; light gray = gyri. (DOCX 2 [file 13229_2016_76_MOESM1_ESM.docx]
